# Predicting the Potential Global Distribution of *Amblyomma americanum* (Acari: Ixodidae) under Near Current and Future Climatic Conditions, Using the Maximum Entropy Model

**DOI:** 10.3390/biology10101057

**Published:** 2021-10-18

**Authors:** Delong Ma, Xinchang Lun, Chao Li, Ruobing Zhou, Zhe Zhao, Jun Wang, Qinfeng Zhang, Qiyong Liu

**Affiliations:** 1 School of Public Health and Health Management, Shandong First Medical University and Shandong Academy of Medical Sciences, Jinan 250117, China; madelong97@163.com (D.M.); lichaoicdc@163.com (C.L.); 2 State Key Laboratory of Infectious Diseases Prevention and Control, National Institute for Communicable Disease Control and Prevention, Chinese Center for Disease Control and Prevention, Beijing 102206, China; lxc960506@163.com (X.L.); zrb9610@126.com (R.Z.); zhezhao@mail.sdu.edu.cn (Z.Z.); wangjun@icdc.com (J.W.); 3 Shandong University Climate Change and Health Center, School of Public Health, Shandong University, Jinan 250012, China

**Keywords:** ticks, *Amblyomma americanum*, species distribution modeling, MaxEnt, climate change

## Abstract

**Simple Summary:**

*Amblyomma americanum* (the lone star tick) is a pathogen vector that bites humans. It can cause severe disease in humans and animals, and may spread as the climate changes. We used a maximum entropy model to predict that the global *Amblyomma americanum* risk area is 3.39 × 10^6^ km^2^. Our work could help tailor related control strategies.

**Abstract:**

*Amblyomma americanum* (the lone star tick) is a pathogen vector, mainly from eastern North America, that bites humans. With global integration and climate change, some ticks that are currently confined to a certain place may begin to spread out; some reports have shown that they are undergoing rapid range expansion. The difference in the potential geographic distribution of *A. americanum* under current and future climatic conditions is dependent on environment variables such as temperature and precipitation, which can affect their survival. In this study, we used a maximum entropy (MaxEnt) model to predict the potential geographic distribution of *A. americanum*. The MaxEnt model was calibrated at the native range of *A. americanum* using occurrence data and the current climatic conditions. Seven WorldClim climatic variables were selected by the jackknife method and tested in MaxEnt using different combinations of model feature class functions and regularization multiplier values. The best model was chosen based on the omission rate and the lowest Akaike information criterion. The resulting model was then projected onto the global scale using the current and future climate conditions modeled under four greenhouse gas emission scenarios.

## 1. Introduction

In the USA, *Amblyomma Americanum* (the lone star tick) is considered to be one of the most aggressive ticks in public health and has veterinary importance [1]. It can be divided into four life stages: egg, larva, nymph and adult. All stages of the tick readily feed on people, companion animals, livestock and wildlife. Ticks can cause host blood loss, skin allergies and other symptoms when they attach to the human body and suck blood, and can transmit *Francisella tularensis* (the agent of tularemia), *Ehrlicia chaffeensis* (the causative agent of human monocytic ehrlichiosis), *Ehrlichia ewingii* (the agent of human granulocytic ehrlichiosis), *Coxiella burnetiid* (the agent of Q fever) [2,3,4,5,6] and *Rickettsia amblyommatis* [7,8]. Lone star tick bites have also been linked to α-gal allergy, which can give rise to a bizarre recurrent life-threatening allergic reaction to red meat [9]. Though it is not apparently a vector of *Borrelia burgdorferi* sensu stricto (the agent of Lyme disease) [10], *A. americanum* has been confirmed to have transmitted the southern tick-associated rash illness (STARI) pathogen, causing a disease clinically similar to Lyme borrelioses [11], which has caused great harm to human health.

*Amblyomma americanum* has a wide distribution along the Atlantic coast of the USA, from New York to Florida and west into Texas and Oklahoma [12]. The core distribution area is located in the southeast of the USA, though there it has had a tendency to expand to the north in recent years; this may be related to climate change [13]. In [14], the authors showed that climate change will affect the growth, development and distribution of ticks, as temperature plays an important role in the lifecycle of ticks. When the temperature is lower than a certain value, the death probability of ticks will increase greatly, whereas an increase in temperature may help the species to be distributed in areas where the climate was previously unsuitable [15].

Modelling is an effective approach to understanding a species’ distribution [16]. In recent years, ecological niche modeling (ENM) is being more and more widely used in the field of species distribution. The model mainly uses the known distribution data and related environmental variables of a species to construct the model according to a certain algorithm, judges the ecological needs of a species, and projects the operation’s results to a different time and space to predict the actual distribution of that species [17]. The Maxent model is one of the more widely used species distribution models. Studies have shown that among the models for predicting species distribution, the Maxent model performs well [18], as it can predict the distribution of suitable habitats for narrow and rare species with a unique natural history, spanning terrestrial and aquatic lifestyles [19]. The Maxent model can avoid commission errors in predicting a species’ distribution [20]. It has been used in many previous studies on a global scale, for species such as *Cydia pomonella* [21], *Dalbulus maidis* [22] and *Bactrocera zonata* [23].

Under global climate change, previously unsuitable areas may become more and more suitable. The models in some similar studies used the species occurrence data with data on the climatic variables and some species characteristics to find the current geographic distribution area and future areas under various climatic scenarios [24,25]. The maximum entropy model is one of the models used to predict current and future potential areas of geographic distribution [26], especially for invasion biology [27], protection of biodiversity [28] and the transmission risks of parasitic diseases [29].

This study aimed to predict the potential global geographic distribution of *A. americanum* and the environment variables affecting the distribution area under near current and future climate scenarios using the maximum entropy model and the Coupled Model Intercomparison Project Phase 6 (CMIP6) data, and to elucidate how this potential distribution of *A. americanum* could change under four climate change scenarios in the future.

## 2. Materials and Methods

### 2.1. The Source and Selection of Occurrence Points

Data were selected from the Global Biodiversity Information Facility, (GBIF; https://www.gbif.org/, accessed on 6 May 2021), Walter Reed Biosystematics Unit, (WRBU; https://www.wrbu.si.edu/, accessed on 6 May 2021), Biodiversity Information Serving Our Nation (BISON; https://bison.usgs.gov/#home, accessed on 6 May 2021) and a literature review, for which the time range was 1990–2020. The distribution points obtained by downloading and consulting were filtered to remove duplicate samples (same sampling point and time) and points with unclear geographic locations. The accuracy of the coordinate points were checked through Google Earth (https://earth.google.com, accessed on 10 May 2021) and some samples with obvious errors in their geographic location coordinates were removed. After the above screening process, we obtained 2705 points for *A. americanum* and the data were saved in .csv format.

### 2.2. Environmental Data Layers

Nineteen bioclimatic variables and 36 climate variables of historical climate data (at a 2.5 min spatial resolution, including minimum temperature, maximum temperature and precipitation in 12 months from 1970–2000 were download from WorldClim v2.1 (http://worldclim.org/version2, released in January 2020). Current and future climate conditions were modelled (at a 2.5 min spatial resolution) under four greenhouse gas emission scenarios: Shared Socio-economic Pathways (SSPs) ssp1-2.6, ssp2-4.5, ssp3-7.0 and ssp5-8.5. We chose the medium-resolution National (Beijing) Climate Center Climate System Model (BCC-CSM2-MR) as the global climate model (GCM). The names and descriptions of the climate data are shown in Table S1.

### 2.3. Geographic Data

The base map of the world was derived from the 1:110 Natural Earth I (http://www.naturalearthdata.com, accessed on 11 October 2021) drawn by the North American Cartographic Information Society (NACIS).

### 2.4. Application Software

MaxEnt software (version 3.4.1) was obtained from https://biodiversityinformatics.amnh.org/, accessed on 11 October 2021. ArcGIS software (version 10.7) was purchased by the Vector Control Department of the Institute of Infectious Disease Control and Prevention, Chinese Center for Disease Control and Prevention. R software (version 4.0.3) was obtained from http://www.r-project.org/ (accessed on 12 May 2021). ActivePrel (version 5.26) was downloaded from https://www.activestate.com/products/perl/downloads (accessed on 12 May 2021).

### 2.5. Data Processing

#### 2.5.1. Selection and Transformation of Distribution Points

The collection of species distribution points can be affected by the degree of accessibility of the species’ distribution area or the bias of the human researcher, resulting in species distribution data in a particular area that is too dense, which may lead to errors in the prediction results. In order to eliminate the impact of this aspect of the data on the prediction results to a certain extent, and reduce the problems related to spatial sampling deviation, we used ENMTools, which is a Prel script with a graphical user interface written with the Tk package to filter the distribution data in the raster of the environment layer [30], and we finally obtained 1695 occurrence points and organized the data into a .csv file according to the data requirements of MaxEnt software.

#### 2.5.2. Screening and Transformation of Environmental Data

BIO1–19, the monthly minimum temperature, monthly maximum temperature, etc. are based on temperature and precipitation, derived from calculations based on different needs. Therefore, the inevitable autocorrelation and multiple linearity between these variables need to be fully considered when considering climate variables. On the basis of their biological significance, some climate variables were removed to reduce the influence of redundant information on the simulation results; alternatively, the dimensionality of the ecological space can be reduced by adjusting the model parameters, and thus the predictability of the model can be increased.

This study used ArcGIS to sample the data at the relevant distribution points of all layers and used R software to perform Pearson correlation analysis of the data to eliminate the influence of collinearity on the model fitting process and interpretation of the results. If the absolute value of the correlation coefficient of a certain pair of variables is greater than 0.8, this indicates that there is a problem of collinearity between the two variables. We initially used 52 layers in the model. Several bioclimatic variables (mean temperature of wettest quarter (BIO8), mean temperature of driest quarter (BIO9), precipitation of warmest quarter (BIO18) and precipitation of driest quarter (BIO19)) were a priori excluded from the WorldClim archive because these layers have been shown to have spatial artefacts that could affect niche modeling [31]. The remaining environmental variables were evaluated regarding their importance, based on their contribution to the distribution of *A. americanum*. Variables with small contribution rates were eliminated, and environmental variables with low biological significance in the group with high correlation were removed from the remaining layers. The dominant environmental variables were obtained as the final climate layer for modeling.

### 2.6. Maximum Entropy Model and Optimization

The Maxent model is preferred by researchers due to the stable results, the short time needed to run the model and its ease of operation [32,33]. The model can be run even if there is only a small amount of occurrence data, and it can test the predicted model [34]. MaxEnt version 3.4.1 was used in the present study. The biggest feature of version 3.4 is the addition of a complementary log–log (Cloglog) output method based on the inhomogeneous Poisson distribution process (IPP), which was used to predict the suitable area for a species.

#### 2.6.1. Selection of Model Parameters

Through a literature review and by combining the biological characteristics of *A. americanum*, all the collected distribution points of the *A. americanum* were imported into the MaxEnt software, set to random seed 75% distribution point modeling and 25% distribution point verification modeling, the output format was “Cloglog”, the output file type was “asc”, the maximum iteration mode was to set select “Bootstrap” and the maximum number of repetitions was 5000. The number of repeated training rounds was set to 10 to reduce the uncertainty caused by outliers by applying threshold rule select “10 percentile training presence”. Finally, the program generated response curves and performed jackknifing to measure each variable’s importance. The regularization multiplier and the combination of features may influence the predictive performance and accuracy of the model [35], so we selected the best model by setting the feature combination (FC) and regularization multiplier (RM). The RM parameter was set to 8 levels: 0.5, 1, 1.5, 2, 2.5, 3, 3.5 and 4, and there were 5 feature combinations: automatic linear (Linear, L), quadratic (Q), fragmentation (hinge, H), product (P) and threshold (T). We manually set 5 characteristic parameters to obtain 8 features (L, LQ, LQP, QHP, LQH, LQHP, QHPT and LQHPT). To get the best model and control overparameterization, the R package “ENMeval” was used. The ENMeval package was implemented in R 4.0.3 “Checkerboard2” to search for the lowest delta value for Akaike’s information criteria corrected for small sample sizes (AICc) to run the MaxEnt software on the candidate models.

#### 2.6.2. Classification of the Potential Suitable Distribution Areas

We used MaxEnt software to predict the current and future potential geographic distribution of *Amblyomma americanum*. The resulting data output of MaxEnt software is in ASCII format. The asc format file in the simulation results obtained after running MaxEnt was the optimized parameters converted into raster data by the Conversion Tools-ASCII to Raster in ArcGIS, and the suitable zones were classified. There are many ways to divide levels in ArcGIS software: manual defaults to 10 levels, which are too finely divided; the range of highly suitable areas divided by the standard deviation is too narrow; the range of highly suitable areas divided by the geometrical interval and quantile is too wide. Based on the inherent natural groupings in the output data of MaxEnt software, this study used the “natural break point classification (Jenks)” classification method in ArcGIS to classify four suitable areas for *A. americanum*: highly suitable areas, moderately suitable areas, less suitable areas and unsuitable areas. Finally, we used ArcGIS to reclassify the layers, and calculate and count the area of each suitable location.

#### 2.6.3. Evaluation of the Model Results

The omission rate provides information on model differences and overfitting. The data were evaluated at a specific threshold to maintain a good model. The ROC curve analysis method was used to test the accuracy of the results of the suitable area. The ROC curve is a curve formed by the true positive rate on the ordinate and the false positive rate on the abscissa. The AUC (area under the ROC curve) value is the area between the ROC curve and the point on the abscissa. The larger the AUC, the greater the distance from the random distribution, and the greater the correlation between the environmental variables and the geographic distribution model of the predicted species, that is, the better the prediction effect of the model. Because the AUC value is not affected by the threshold, the evaluation is more objective [36,37]. The evaluation standard of a ROC curve is: AUC value = 0.5~0.6, failure; 0.6~0.7, poor; 0.7~0.8, fair; 0.8~0.9, good; 0.9~1.0, excellent.

#### 2.6.4. Predicting the Future Potentially Suitable Distribution Areas

We used MaxEnt software to analyze the suitable areas of four SSPs (ssp1-2.6, ssp2-4.0, ssp3-7.5 and ssp5-8.5) in the years (2021–2040, 2041–2060, 2061–2080 and 2081–2100) for *A. americanum* to make predictions. Combined with the distribution points and dominant environmental data of *A. americanum*, the corresponding environmental data layers of ssp1-2.6, ssp2-4.0, ssp3-7.5 and ssp5-8.5 were exported to MaxEnt, and the parameter settings and other operations were the same as those described in Section 2.6.1, Section 2.6.2, Section 2.6.3.

## 3. Results

### 3.1. The Major Parameters of the Maximum Entropy Model

**Figure 1:**
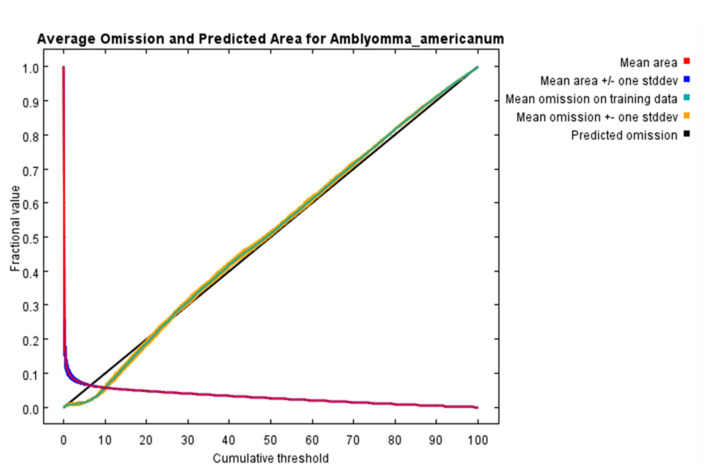
The output of the Maxent model’s for the training omission rate and predicted area as a function of the cumulative threshold.

We applied ENMTools analysis to all collected occurrence points and finally obtained 1695 occurrence points of *A. americanum* for the maximum entropy model. After jackknife analysis was applied to select bioclimatic and environment variables, the variables finally selected are shown in Table 1. The future combinations and regularization multiplier of the maximum entropy model in this study were LQH and 2. In the Maxent model, it can be seen from the contribution of each variable to the distribution of *A. Americanum* (Table 1) that under the near current climatic conditions, the mean omission curve of the test data has a slight deviation near the predicted omission (Figure 1). The mean AUC under the current climatic conditions of the Maxent model is 0.970, and the standard deviation is 0.001.

### 3.2. Global Distribution Points of Amblyomma americanum

**Figure 2:**
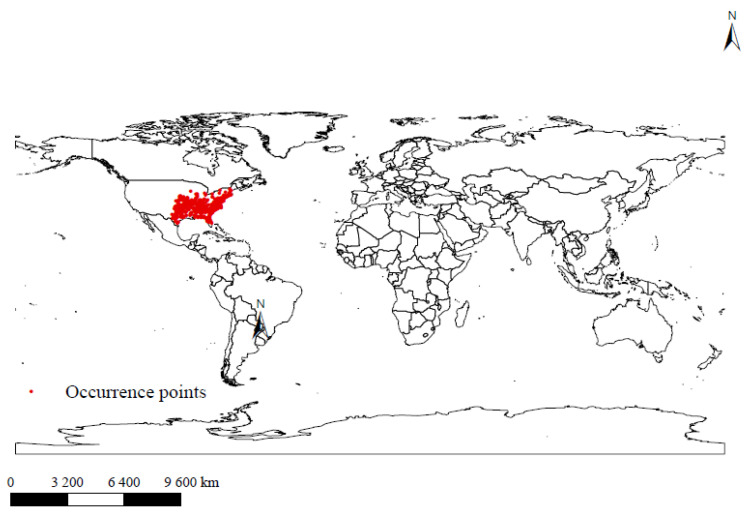
Current occurrence and global distribution of *A. americanum*.

It can be seen from Figure 2 that *A. americanum* is relatively concentrated in the United States, located in the eastern and southern regions of the eastern United States, and this area is a highly suitable area for *Amblyomma americanum*.

### 3.3. The Potential Distribution of A. americanum under Near Current Climatic Conditions

**Figure 3:**
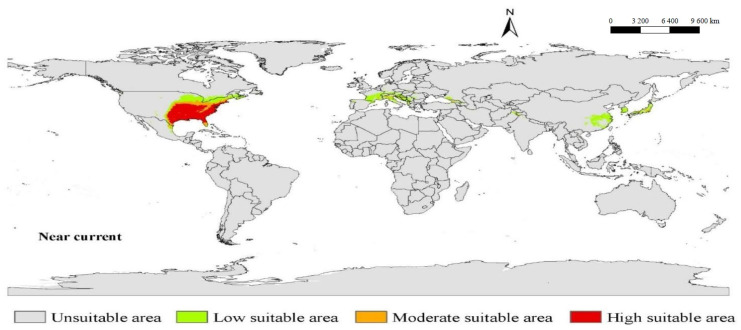
Predicted area suitability under near current climatic conditions. The predicted potential geographic distribution of *A. americanum* is not only in the current occurrence area.

The potential geographic global distribution of *A. americanum* under the near current climatic conditions was based on 10 percentile training with a Cloglog presence threshold. The lowest presence threshold of this model was 0.0263. We used ArcGIS 10.7 Desktop to reclass the potentially suitable areas in MaxEnt’s .asc format output, and used natural breaks (Jenks) to reclassify areas as unsuitable areas, less suitable areas, moderately suitable and highly suitable areas (Figure 3). Under the current climatic conditions, the suitable areas for *A. americanum* in the world include the eastern and southern parts of the USA, the Bahamas, the southeast of Canada and the northeast of Mexico in North America; Switzerland, the southern part of France, Italy, Austria, Germany, Andorra, Croatia and Serbia in Europe; and the southeastern part of China, South Korea and Japan in Asia.

### 3.4. The Relationship between the Distribution of A. americanum and the Environmental Variables

**Figure 4:**
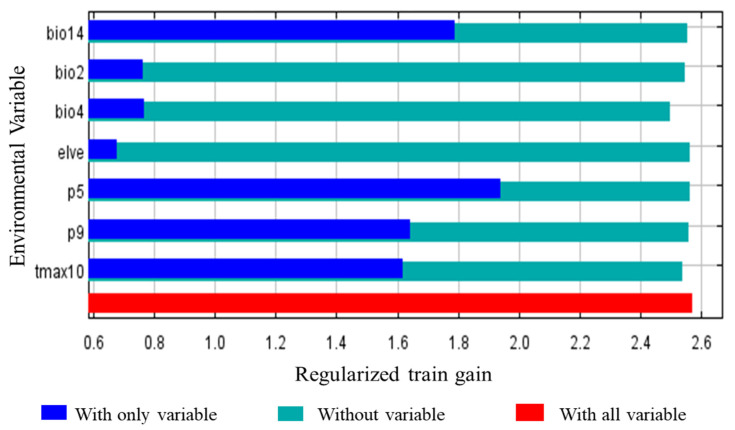
Jackknife analysis results showing the environmental variables and regularized training gain. Prec5 significantly contributed to the suitability of the areas of *A. americanum* and Bio4 has the most information that other environmental variables do not have.

**Figure 5:**
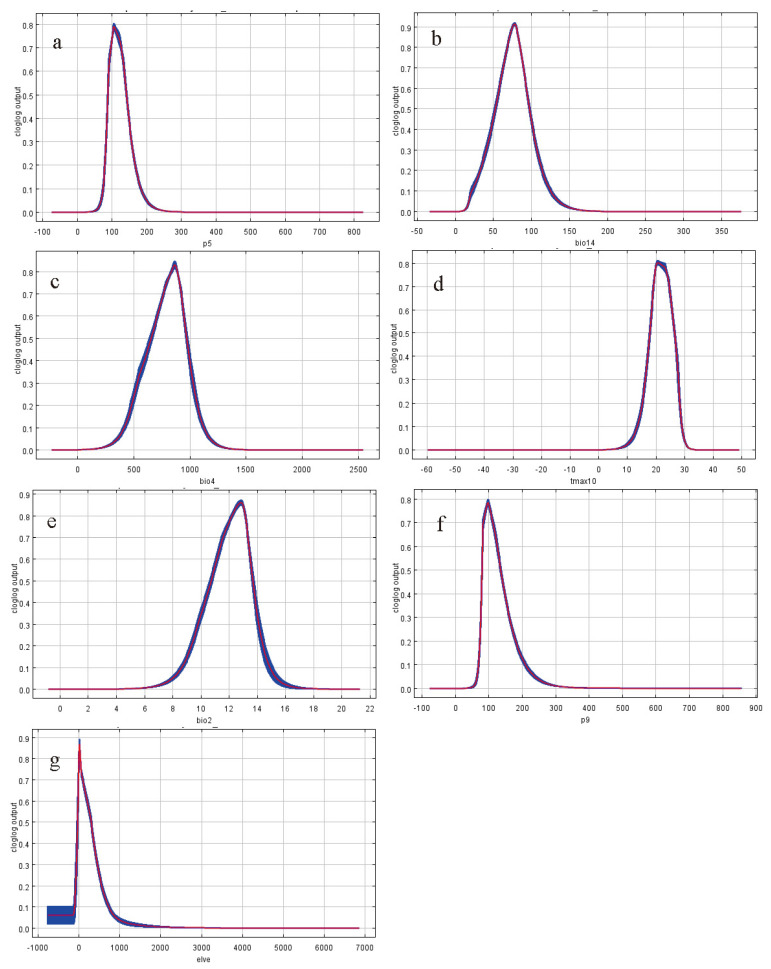
Response curves of environmental variables to the distribution probability of *A. americanum*: (**a**) Precipitation in May, (**b**) precipitation of the driest month, (**c**) temperature seasonality (standard deviation × 100), (**d**) monthly average maximum temperature in October, (**e**) mean diurnal range (mean of monthly (max temp–min temp)), (**f**) precipitation in September and (**g**) elevation. Curves shown are averages over 20 replicate runs; blue margins show the ±SD calculated over 20 replicates.

We used the jackknife method to choose the most important variables that impact the distribution of *A. americanum*. Every environmental variable has two gains, marked as different lengths and colors. The long bar shows the influence on or contribution to the distribution and the short bar shows the information that other variables do not have (Figure 4). The results show that the suitable precipitation in May ranged between 55 and 215 mm, and the most suitable value is 105 mm, with a trend of increasing first and then decreasing (Figure 5a). The suitable precipitation of the driest month ranged between 10 and 500 mm, and the most suitable value was 75 mm, with 10 mm to 75 mm being able to increase the survival probability of *A. americanum* (Figure 5b). The suitable temperature seasonality ranged between 203 and 1380 (Figure 5c). The monthly average maximum temperature in October ranged between 8 and 32 °C, and the most suitable value was 20 °C. The probability of occurrence increased with increasing temperature when the temperature was between 8 and 20 °C, and the probability of occurrence decreased with increasing temperature when the temperature was between 20 and 30 °C (Figure 5d). The suitable mean diurnal range ranged between 6.1 and 17.2 °C, and the most suitable value was 12.8 °C (Figure 5e). The suitable precipitation in September ranged between 60 and 305 mm, and the most suitable value was 100 mm, with the same trend as Prec5 (Figure 5f). The suitable elevation ranged between −86 and 1000 m, and the most suitable value trended to 0 m (Figure 5g). Thus, the temperature and precipitation that can affect the survival probability of *A. americanum* are different in different months.

### 3.5. The Range of Suitable Areas for A. americanum under Future Climatic Conditions

**Figure 6:**
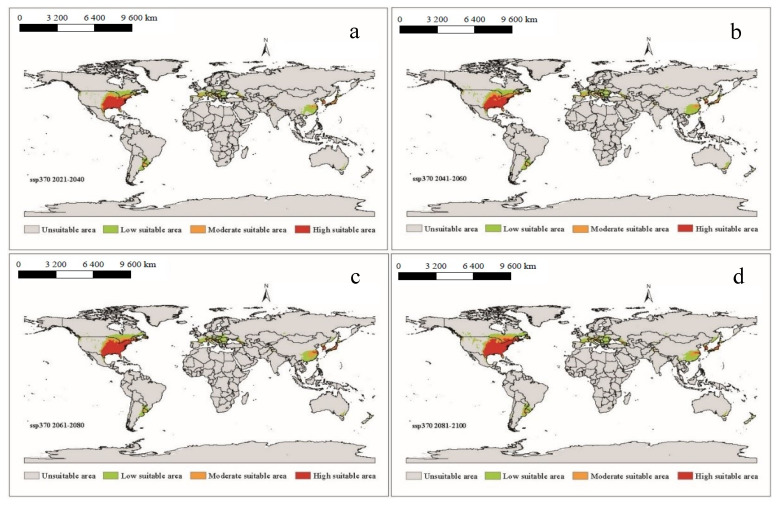
The potential distribution of suitable areas for *A. americanum* around the world under the climatic conditions of shared socio-economic pathway 1–2.6 (sustainability and radiative forcing of 2.6 W/m^2^ to 2100) during different periods of the 21st century: (**a**) 2021–2040, (**b**) 2041–2060, (**c**) 2061–2080 and (**d**) 2081–2100.

**Figure 7:**
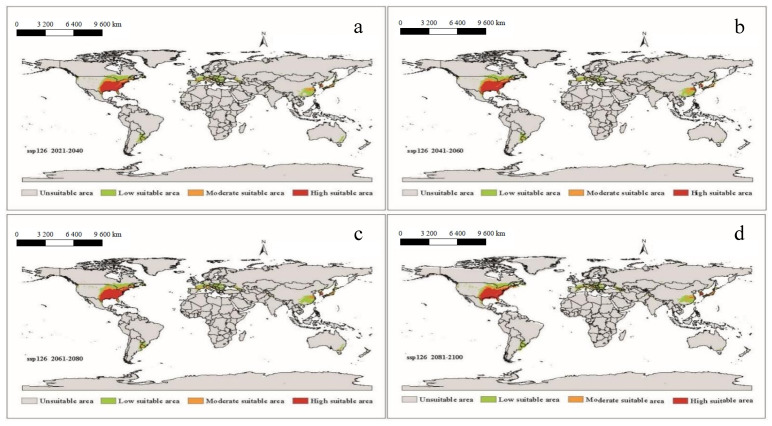
The potential distribution of suitable areas for *A. americanum* around the world under the climatic conditions of shared socio-economic pathway 2–4.5 (middle of the road and radiative forcing of 4.5 W/m^2^ to 2100) during different periods of the 21st century: (**a**) 2021–2040, (**b**) 2041–2060, (**c**) 2061–2080, (**d**) 2081–2100.

**Figure 8:**
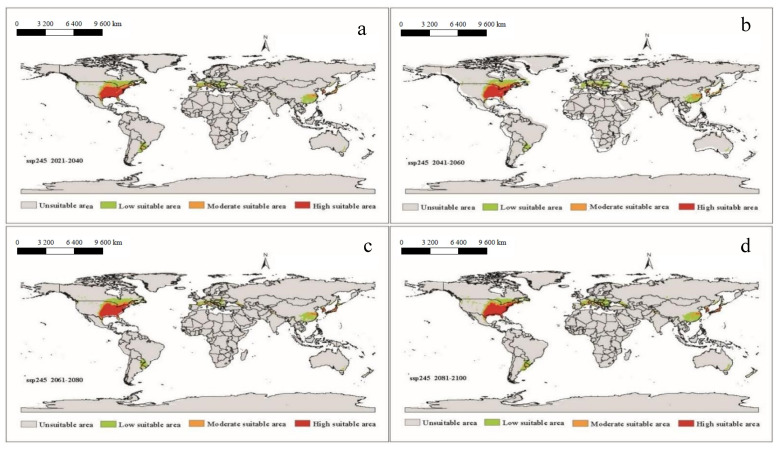
The potential distribution of suitable areas for *A. americanum* around the world under the climatic conditions of shared socio-economic pathway 3–7.0 (regional rivalry and radiative forcing of 7.0 W/m^2^ to 2100) during different periods of the 21st century: (**a**) 2021–2040, (**b**) 2041–2060, (**c**) 2061–2080, (**d**) 2081–2100.

**Figure 9:**
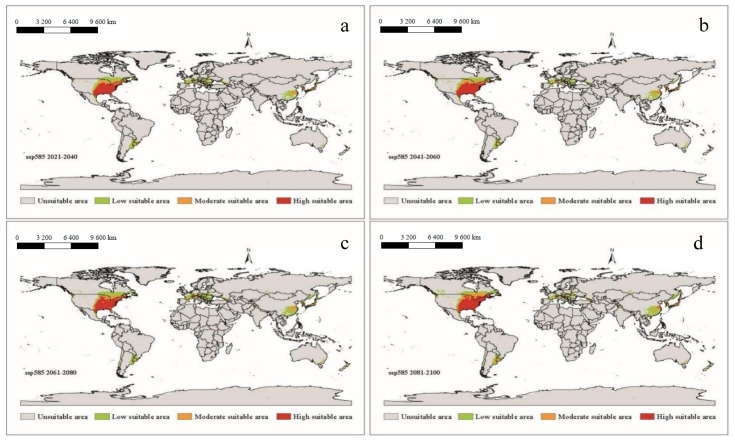
The potential distribution of suitable areas for *A. americanum* around the world under the climatic conditions of shared socio-economic pathway 5–8.5 (fossil-fueled development and radiative forcing of 8.5 W/m^2^ to 2100) during different periods of the 21st century: (**a**) 2021–2040, (**b**) 2041–2060, (**c**) 2061–2080, (**d**) 2081–2100.

It can be seen that under future climatic conditions from 2021 to 2100, the suitable areas for *A. americanum* in the world are similar to the near current distribution, but the phenomenon of spreading will have taken place (Figure 6, Figure 7, Figure 8 and Figure 9). According to the prediction results of the Maxent model, during the period 2021–2100, the distributions of suitable areas for *A. americanum* under the four climate scenarios of 1–2.6, 2–4.5, 3–7.0 and 5–8.5 are larger than the near current distribution of suitable areas, and the area change rate ranges from 63.42% to 117.11% (Table 2). The potentially less suitable areas have a significant increase under all four climatic conditions, and the moderately suitable areas and highly suitable areas have a relative change. The total suitable areas change from 2.15 (×10^6^ km^2^) to 3.97 (×10^6^ km^2^), and the change under ssp3-7.0 and ssp5-8.5 is greater than that under than ssp1-2.6 and ssp2-4.5. We think that the ssp2-4.5 scenario is the closest to the existing policy scenario, and the maximum area change is 100.59% compared with the current climatic conditions (Table 2). We can see from the trend lines of the less suitable areas, the moderately suitable areas and the highly suitable areas that all trends increase with the passage of time (Figure S1).

## 4. Discussion

In this study, we used the Maxent model to predict the suitable areas for lone star ticks under current climatic conditions and future climatic conditions. We found that under the current and future climatic conditions, the potential distribution of the suitable habitat range of *A. americanum* will expand to the north. Our results are similar to those of Sagurova [13], with the main limitation variable that led to Canada not being a suitable habitat being the minimum temperature. However, with climate change, the temperature in the cold areas may reach conditions in which *A. americanum* could survive. The temperature changes in the south and west did not reach the maximum temperature at which *A. americanum* can survive.

The suitable areas predicted by MaxEnt in the current study are wider than the present distribution of *A. americanum* under current and future climate conditions. The reason for this result is that the independent variables lack some variables such as vegetation type, host distribution area and so on. As we all know, the hosts and vegetation cover rate are the influencing factors for ticks. The model fitted does not consider the influence of these on *A. americanum*, and therefore it can lead to small overpredictions for the near current condition and future climate conditions. For example, it will predict expansion northward when the temperature and precipitation are right for *A. americanum* survival. Work on global economic and quarantine measures for *Amblyomma americanum* are urgent, due to the expansion of its potential distribution range, especially across Asia, North America, and Europe. We can see that a large number of suitable areas are located in China, possibly because of the similar climate in southern China and the eastern USA: they have the same Köppen climate classification: Cf (warm temperate climate, fully humid). *A. americanum* is unlikely to arrive in China in a non-artificial way because these ticks need a host or vehicle to transport them. This may be the reason why the ticks are confined to the United States. Under climate change, the areas with changed temperatures and precipitation are expected to either increase or decrease the suitable area for *A. americanum*. Our future potential distribution results show that climate change would affect the geographic distribution area of lone star ticks, especially in less suitable habitats. Our results also suggest that there would be an increase in all suitable areas with low, moderate, and high suitability in response to global warming. Our results are in agreement with other results, but may be slightly different, such as those on *Amblyomma* ticks [38]. Pascoe found that there were suitable areas for *A. americanum* in California [39], but Raghavan found in a simulation of the suitable areas for A. *americanum* in North America that *A. americanum* was largely restricted to the east and central areas of the USA [40]. *A. americanum* and other ticks are still threatening invasion around the globe; for example, *Amblyomma* spp. are listed as notifiable under the New Zealand Biosecurity (Notifiable Organisms) order 2016. With climate change, it is more and more important for countries in Asia and Europe to impose comprehensive quarantine measures.

The results of the MaxEnt model suggest that temperatures and precipitation are the most important variables affecting the potentially suitable areas for *A. americanum* among all the environmental variables. Seven bioclimatic variables were selected in the predictive model we fitted. From the results of the jackknife test of variable importance, we found that the environmental variable with the highest gain when used in isolation was Prec5, which therefore appears to have the most useful information by itself. The environmental variable that decreased the gain the most when it was omitted is BIO4, which therefore appears to have the most information that is not present in the other variables. Through screening the correlation analysis of each variable, we deleted some variables, such as Tmin12, Tmin1 and Tmin2, due to their high correlation with BIO4, which made a relatively high percent contribution. This is more consistent with the results of other studies [39]. The early-season activity of adult and nymphal ticks precedes that of larvae [12], with some larvae possibly overwintering in Georgia. Of the variables that were selected, only temperature seasonality (standard deviation × 100) (BIO4) had the most information because the minimum temperature of the coldest month (BIO6), and the minimum temperature of January, February and December had high correlation with BIO4 (absolute value of the correction coefficient ≥ 0.8). Most previous studies have been aimed at a certain country or city, such as New Zealand, Kansas or the continental United States, but our study displayed suitable areas around the whole world. We used the 2.5′ raster climate data from WorldClim version 2.1 to predict the suitable area (this version was released in January 2020) and the future climate data were based on the CMIP6 Shared Socio-economic Pathways, and thus the data’s accuracy is more reliable than that of previous versions [41]. Compared with previous studies, we also predicted suitable areas in the North and South Islands of New Zealand, but the predicted model showed lower risks than Raghavan et al. [13]. Raghavan et al. used the same method for modeling in New Zealand but the fitted model was transferred to four General Circulation models compared with one in our study. The future climate model also raised the possibility of northward range expansion into all eastern and central provinces of Canada, compared with Sagurova et al. [42]. We think there will be hardly any westward expansion, mainly because there will not be enough precipitation. Although the main influencing factors are different, our results are basically consistent with those of Springer et al. [43] and Raghavan et al. [44]. Unlike Springer et al. [43], we did not use growing degree-days, vapor pressure or the number of days with snow cover. Raghavan et al. [44] used the environment variables from Climond, which include data on soil moisture estimates.

According to the existing historical records of *Amblyomma americanum*, its distribution center is in the south and southeast of the eastern United States, but it also occurs in the far north of the United States, especially along the Atlantic coast and the Midwest, and its distribution tends to move northward [45]. This phenomenon may be related to changes in the climate and environment and changes in the distribution of its hosts. The growth and development of ticks are closely related to climate factors such as temperature, humidity, rainfall and photoperiod.

Our research also has some limitations: in the ENM, we fitted only some selected environment variables, such as temperature, precipitation and elevation; soil moisture, host and vegetation type were not included in the analysis, which could be the next step to carry out regarding this issue.

## 5. Conclusions

We found that the Maxent model predicted potentially suitable geographic areas of *A. americanum* under near current and future climatic conditions based on the existing distribution of *A. americanum*, which may enable the precise identification of important environmental variables driving the current and future potential geographic distribution of *A. americanum*, and this was derived from temperature and precipitation as they are the important variables affecting the survival probability. Under future climatic conditions, the total suitable area will be increased and the distribution area will expand. With global warming, some countries will become suitable areas even if they are not currently suitable areas. With global economic integration, *A. americanum* will have more opportunities to be carried to many countries. This is of great guiding significance for improving the awareness of prevention in areas where *A. americanum* is at risk of invasion, thus providing a theoretical basis for early prevention and control in these areas.

## Figures and Tables

**Table 1:** Environmental variables used for predicting the potential geographic distribution of *A. americanum*.

Variables	Description	Unit	Contribution (%)
Prec5	Precipitation in May	mm	41.9
BIO14	Precipitation of the driest month	mm	30.6
BIO4	Temperature seasonality (standard deviation × 100)	\	16.8
Tmax10	Temperature in October	°C	4.5
BIO2	Mean diurnal range (mean of monthly (max temp–min temp))	°C	4.3
Prec9	Precipitation in September	mm	1.2
Elevation	Elevation	m	0.8

**Table 2:** Current and future suitable area for *Amblyomma americanum* across the world under different climatic conditions (×10^6^ km^2^).

Climate Scenario	Period	Less Suitable Areas	Moderately Suitable Areas	Highly Suitable Areas	Total Area	Area Change	Area Change Ratio (%)
current	1970–2000	1.66	0.49	1.24	3.39	0.00	
ssp1-2.6	2021–2040	3.29	0.98	1.56	5.83	2.44	71.98
	2041–2060	2.84	1.03	1.67	5.54	2.15	63.42
	2061–2080	3.18	1.04	1.64	5.86	2.47	72.86
	2081–2100	3.11	1.07	1.81	5.99	2.60	76.70
ssp2-4.5	2021–2040	3.23	1.10	1.60	5.93	2.54	74.93
	2041–2060	3.17	1.15	1.84	6.16	2.77	81.71
	2061–2080	3.06	1.06	1.92	6.04	2.65	78.17
	2081–2100	3.67	1.13	2.01	6.81	3.41	100.59
ssp3-7.0	2021–2040	3.06	1.01	1.56	5.63	2.24	66.08
	2041–2060	3.56	1.27	1.69	6.52	3.13	92.33
	2061–2080	3.32	1.22	2.11	6.66	3.26	96.17
	2081–2100	3.77	1.29	2.31	7.37	3.97	117.11
ssp5-8.5	2021–2040	3.13	1.11	1.71	5.95	2.56	75.52
	2041–2060	3.01	1.19	1.80	6.00	2.61	76.99
	2061–2080	3.45	1.28	1.90	6.63	3.24	95.58
	2081–2100	3.38	1.62	2.14	7.14	3.75	110.62

## Data Availability

Bioclimatic data used in this study can be downloaded at https://www.worldclim.org/, accessed on 11 October 2021. Occurrence records used in this study can be downloaded at https://www.gbif.org, accessed on 11 October 2021, https://www.wrbu.si.edu/ and https://bison.usgs.gov/#home, accessed on 11 October 2021.

## Supplementary Materials

The following are available online. https://www.mdpi.com/article/10.3390/biology10101057/s1. Table S1. Climate variables used in predicting the potential geographic distribution of *A. americanum*. Figure S1. The change in area (×10^6^ km^2^) of the predicted potential distribution of *A. americanum* around the world under future climate conditions during different periods of the 21st century under four shared social-economic pathways climate conditions, compared with the potential distribution area under near current climate conditions: (a) ssp1-2.6, (b) ssp2-4.5, (c) ssp3-7.0, and (d) ssp5-8.5.
