# Supplementary material for: Predicting the Potential Global Distribution of Amblyomma americanum (Acari: Ixodidae) under Near Current and Future Climatic Conditions, Using the Maximum Entropy Model
Source: Biology (Basel). 2021 Oct 18;10(10):1057. doi: 10.3390/biology10101057 (PMC8533137; doi:10.3390/biology10101057)
Supplement: Supplementary file 1 [file biology-10-01057-s001.zip › supplement table&figure.pdf]

Table S1. Climate variables used in predicting the potential geographic distribution of *A. americanum.*

Code	Climate Variables
BIO1	Annual Mean Temperature
BIO2	Mean Diurnal Range(Mean of monthly(max temp – min temp))
BIO3	Isothermality(BIO2/BIO7)(×100)
BIO4	Temperature Seasonality(standard deviation×100)
BIO5	Max Temperature of Warmest Month
BIO6	Min Temperature of Coldest Month
BIO7	Temperature Annual Range(BIO5 – BIO6)
BIO8	Mean Temperature of Wettest Quarter
BIO9	Mean Temperature of Driest Quarter
BIO10	Mean Temperature of Warmest Quarter
BIO11	Mean Temperature of Coldest Quarter
BIO12	Annual Precipitation
BIO13	Precipitation of Wettest Month
BIO14	Precipitation of Driest Month
BIO15	Precipitation Seasonality (Coefficient of Variation)
BIO16	Precipitation of Wettest Quarter
BIO17	Precipitation of Driest Quarter
BIO18	Precipitation of Warmest Quarter
BIO19	Precipitation of Coldest Quarter
Tmin1-12	Monthly Average Minimum Temperature
Tmax1-12	Monthly Average Maximum Temperature
Prec1-12	Monthly Total Precipitation
Elevation	Elevation data

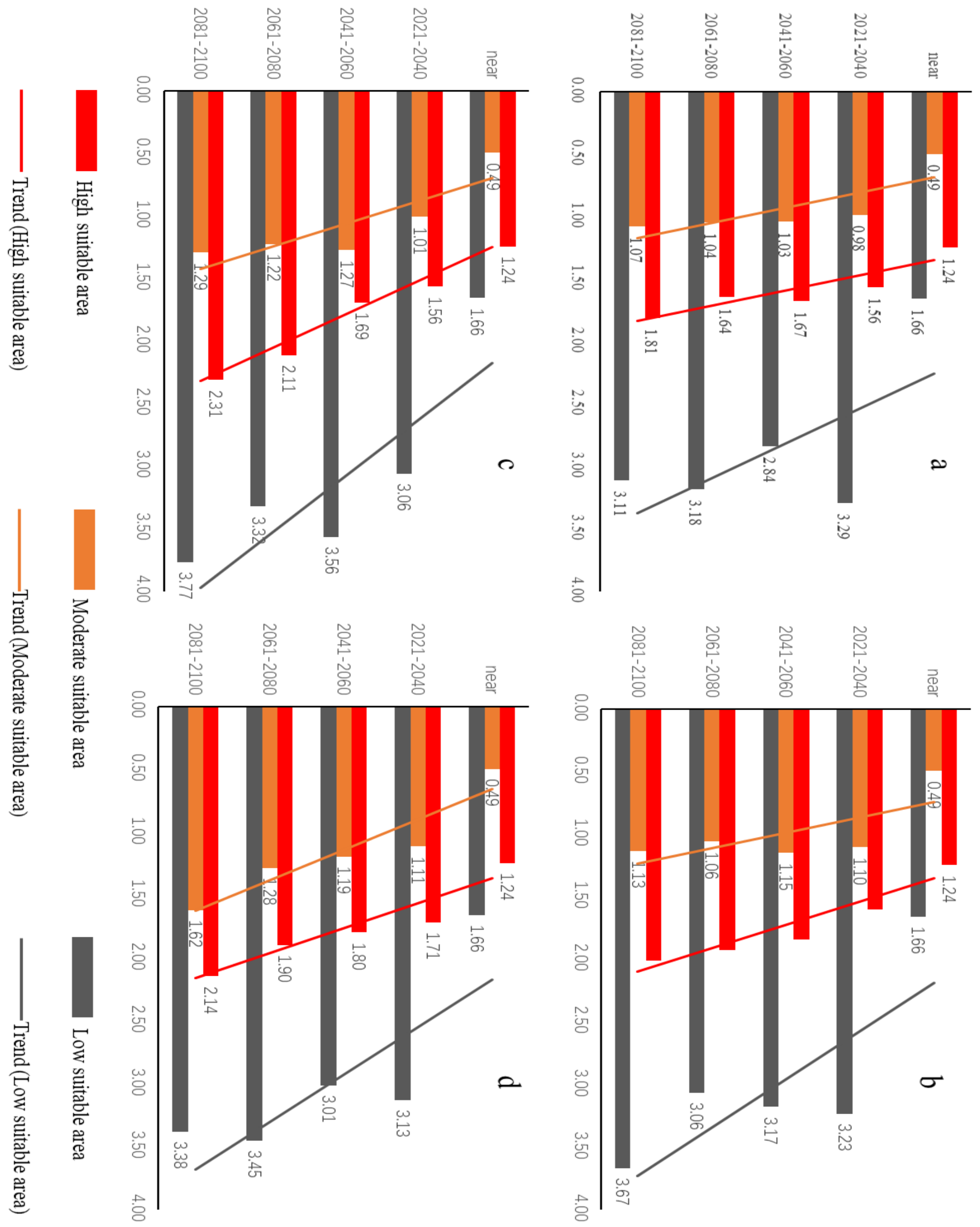

Figure S1. The area change (×10^6 km^2) of predicted potential distribution of *A. americanum* around the world under the future climate conditions during the periods of the 21st century under four shared social-economic pathways climate conditions, compared with the potential distribution area under the near current climate condition and four shared socio-economic pathways: (**a**) ssp1-2.6; (**b**) ssp2-4.5; (**c**) ssp3-7.0; (**d**) ssp5-8.5.
